# Hematology and plasma biochemistries in the Blanding’s turtle (*Emydoidea blandingii*) in Lake County, Illinois

**DOI:** 10.1371/journal.pone.0225130

**Published:** 2019-11-15

**Authors:** Lauren E. Mumm, John M. Winter, Kirsten E. Andersson, Gary A. Glowacki, Laura A. Adamovicz, Matthew C. Allender

**Affiliations:** 1 Wildlife Epidemiology Laboratory, College of Veterinary Medicine, University of Illinois, Urbana, IL, United States of America; 2 Lake County Forest Preserves, Libertyville, IL, United States of America; Oregon State University, UNITED STATES

## Abstract

Chelonians are one of the most imperiled vertebrate taxa on the planet due to changes in the environment, anthropogenic influences, and disease. Over the last two decades, conservation strategies including nest protection, head-starting and meso-predator control have been successfully adopted by the Lake County Forest Preserve District for a population of state-endangered Blanding’s turtles (*Emydoidea blandingii*) in Illinois. Only recently have efforts expanded to assess the effects of management action on turtle health. The objectives of this study were to 1) establish reference intervals for 16 hematologic and plasma biochemical analytes in free-ranging Blanding’s turtles, 2) characterize demographic and temporal drivers of clinical pathology values including age class, sex, month, and year, and 3) describe bloodwork differences between a managed (SBCP) and unmanaged (IBSP) study site. Hematology and plasma biochemistries were performed for 393 turtles from 2017–18 at two sites in the Lake Plain region. Subject or population-based reference intervals were established based on the index of individuality per American Society for Veterinary Clinical Pathology guidelines. Analytes differed by age class [packed cell volume (PCV), total solids (TS), total white blood cell counts (WBC), heterophils, lymphocytes, heterophil:lymphocyte ratio (H:L), total calcium (Ca), calcium:phosphorous (Ca:P), bile acids (BA), aspartate aminotransferase (AST)], sex [H:L, Ca, phosphorus (P), Ca:P, creatine kinase (CK)], month [eosinophils, H:L, Ca, P, uric acid (UA), AST], and year [PCV, WBC, lymphocytes, basophils, H:L, Ca, P, UA]. Several analytes also varied by site [PCV, TS, monocytes, eosinophils, P, UA, AST], suggesting that health status may be affected by habitat management or lack thereof. The results of this study provide a baseline for ongoing health assessments in this region as well as across the Blanding’s turtle range.

## Introduction

The Blanding’s turtle (*Emydoidea blandingii*) is a semi-aquatic, freshwater species inhabiting the Great Lakes and Atlantic Northeast regions of North America [1]. In such biodiverse environments, reptiles play an important energetic role in ecosystem homeostasis, functioning as predators, prey, and commensal species [2]. Due to habitat destruction, road mortality, poaching [3], and mesopredator abundance [4], the Blanding’s turtle has been listed as state-endangered in Illinois, and threatened on the global scale [4]. Because freshwater turtles have great longevity and hold high trophic positions [5], previous studies in Blanding’s turtles have focused on their utility as environmental sentinels. Nevertheless, there is a lack of information characterizing the general health of this imperiled species [6–9].

The largest known Blanding’s turtle populations in Illinois reside in the northern half of the state, including in the Chiwaukee Illinois Beach Lake Plain. Since 2004, the Lake County Forest Preserve District (LCFPD) has performed regular population monitoring on Blanding’s turtles in response to population declines at a specific site of the Lake Plain, Spring Bluff-Chiwaukee Prairie (SBCP). Additionally, in 2010, a Blanding’s Turtle Recovery Program was formed to conserve and recover a sizable Blanding’s turtle population in Lake County [10]. The program has most recently focused on meso-predator control, head-starting juvenile turtles, and restoration of both aquatic and upland habitats functioning to ensure hatching and initial growth prior to release [10–11]. LCFPD’s Blanding’s Turtle Recovery Program has been successful in increasing juvenile survivorship, recruitment and the overall population size at SBCP, however health assessment has not been performed [10]. In 2017, LCFPD extended their ecological assessments to a second Lake Plain location, Illinois Beach State Park (IBSP), in which future conservation management similar to that of SBCP may be carried out if so indicated. This expansion has provided a unique opportunity to integrate health assessment differences that reflect conservation interventions at both a managed (SBCP) and unmanaged site (IBSP).

Clinical pathology monitoring provides an objective assessment of overall health status in free-ranging turtle populations [12]. Establishing reference intervals allows for assessment of deviations from baseline and can serve as a guide in identifying population stressors to inform management practices [13]. Previous studies regarding clinical pathology in wild reptiles have found significant differences in hematologic and biochemical parameters based on age, sex, and season, thus it is important to define and discuss significant differences in terms of demographics and temporality [13–19]. The objectives of this study were to (1) establish reference intervals for hematology and plasma biochemistries in free-ranging Blanding’s turtles, (2) characterize demographic and temporal drivers of clinical pathology values including age class, sex, month, and year, and (3) evaluate differences in bloodwork values between a site with ongoing management practices (SBCP) and an unmanaged site (IBSP) to assess the utility of clinical pathology for informing conservation intervention strategies.

## Materials and methods

### Study sites and capture methods

Sampling of Blanding’s turtles was performed in May-August, 2017 and 2018 at Spring Bluff-Chiwaukee Prairie (SBCP) and Illinois Beach State Park (IBSP). Turtles were captured with the aid of radiotelemetry, hoop traps, or by visual encounter. All telemetered individuals were adults. Those from SBCP had been previously tracked for up to ten years. Those from IBSP initially received tracking devices in 2017. Hoop traps were placed in marsh waters that were characteristic of Blanding’s habitat or previously had Blanding’s turtles found in or near them, and were checked every 24 hours. The sample population included both wild-born and head-started individuals from the Blanding’s Turtle Recovery Program. All individuals were marked with a unique shell notch and were released at site of capture the same day [20]. All procedures were approved by the University of Illinois Institutional Animal Care and Use Committee (Protocols: 18000 and 18165) and under approval from the Illinois Department of Natural Resources Endangered and Threatened (Permits: 14–046 and 1042).

### Physical examination and sample collection

Upon capture, each turtle was assigned a permanent ID. Mass, sex, age class, and general physical exam observations were recorded, including visual appearance of the eyes, nose, oral cavity, ears, legs, digits, shell, integument, and cloaca. Each individual was characterized as juvenile (<250 grams), sub-adult (250–750 grams), or adult (>750 grams). Blanding’s turtles were deemed sexually mature at >750 grams by the LCFPD based on historical observation of the youngest fertile female. Sex was classified as male, female or unknown. Sex of head-started turtles was known based on incubation temperature [21]. Sex of adults was determined by plastron concavity, males being more concave [1], and precloacal distance, cloaca extending up to or past the carapacial edge in males [22]. Wild-born individuals were classified as unknown when a confident determination could not be made based on external characteristics.

Whole blood was collected from the subcarapacial sinus via 22-gauge or 25-gauge needle. No more than 0.6% of body weight, limited to 3 mL, of whole blood was drawn and placed into lithium-heparinized collection tubes (BD microtainer, Jorgensen Laboratories Inc, Loveland CO 80538). Blood samples were placed on ice in a cooler until returning to the lab each afternoon to perform sample analysis.

### Clinical pathology

White blood cell counts (WBC), packed cell volume (PCV), total solids (TS), and blood smears were performed within 12 hours of sample collection. Plasma separation for biochemistry analysis was performed within 8 hours of venipuncture by centrifugation (6,000 rpm x 10 minutes). Plasma samples were frozen at -20°C for up to four months prior to analysis.

PCV and TS were performed by filling two sodium heparinized microhematocrit tubes (Jorgensen Laboratories, Inc., Loveland, CO 80538) from one LH microtainer tube. Each sample was centrifuged (5,000 rpm x 5 minutes) and the PCV recorded. Total solids were determined with a hand-held refractometer (Amscope RHC-200ATC refractometer, National Industry Supply, Torrance, CA, USA) using plasma from the microhematocrit tubes. WBC counts were determined using an Avian Leukopet (Vet Lab Supply, Palmetto Bay, FL, USA) on a Bright-line hemacytometer (Hausser Scientific, Horsham, PA, USA) following the manufacturer’s protocol. Fresh blood smear slides were stained with a modified Wright’s Giemsa stain and one hundred white blood cell differential counts were performed by a single observer (LM). Heterophil:Lymphocyte (H:L) ratio was calculated by dividing heterophil values by lymphocyte values.

Biochemistry profiles were performed on plasma samples by the University of Illinois’ Veterinary Diagnostic Lab (AU680 Chemistry System, Beckman Coulter, Brea, CA 92821 USA). The following blood values were evaluated: calcium (Ca), phosphorous (P), bile acids (BA), uric acid (UA), creatine kinase (CK) and aspartate aminotransferase (AST). Calcium:Phosphorus (Ca:P) ratio was calculated by dividing calcium values by phosphorus values. Parameters in this study were selected based on utilization in historical chelonian clinical pathology studies.

### Reference intervals

Inclusion criteria for the reference interval (RI) portion of the study required a physical examination lacking clinically significant abnormalities and sample quality free of hindrance (clotting, hemolysis, lymph contamination). Exclusion criteria included missing values, the presence of clinically significant physical examination abnormalities, and poor sample quality. Outliers were identified using statistical software (MedCalc, Belgium), following methods previously described [23], and excluded. Reference intervals were then generated according to American Society for Veterinary Clinical Pathology guidelines for subject-based RI [24–25] and population-based RI [26–27].

Subject-based RI were generated from longitudinally-sampled turtles. Initially, the inter-individual variation (CV_g_) and intra-individual variation (CV_i_) was determined for each CBC and chemistry parameter. Analytical variation (CV_a_) is unknown for most parameters and was not determined in this study, but values from previous reports in dogs [27], birds [28] or box turtles (unpublished) were used when available. The index of individuality was calculated as (*CVi*+*CVa*)/*CVg* for parameters that have a published CV_a_ and as *CVi*/*CVg* for parameters without a known analytical variation. Reference change values (RCV) were also calculated using 1.96×2^1/2^×*CVi* [24]. RCV is more robust to deviations between individual samples [26]. If the index of individuality was less than 0.6, a subject-based RI was calculated, and if greater than 0.6 a population-based RI was created [24]. Population based RI were then calculated using the parametric method. Ninety percent confidence intervals of each reference interval bound were determined using bootstrapping, per ASVCP guidelines.

### Statistical analysis

Statistical analysis was performed for combined data from 2017 and 2018 to give a complete two-year picture of population health. Statistical modeling was performed for analytes determined to be best represented by a population-based RI, and analysis included results from all samples. For analytes best represented by subject-based RI, linear mixed modeling with turtle ID as a random effect was performed on results from individuals with multiple samples, while general linear modeling was performed on results from individuals sampled only once (similar to population-based RI analytes).

Descriptive statistics (mean, median, standard deviation, minimum and maximum) were tabulated for continuous variables (hematologic, plasma biochemical analytes). Normality was assessed using the Shapiro-Wilk test, Q-Q plots, skewness, and kurtosis. Normally distributed data were compared between sexes and sites using an independent samples t-test and between age classes using an analysis of variance (ANOVA). When data were not normally distributed, a Mann-Whitney U test or a Kruskal-Wallis one-way analysis of variance was used, respectively. Descriptive statistics and univariate analyses were performed using SPSS (Version 24, IBM Statistics, Chicago, IL) and alpha levels were set at p = 0.05.

Following univariate analyses, we constructed a series of general linear models with each clinical pathology variable as a dependent variable, and age class (adult, subadult, juvenile), sex (male, female, unknown), month (May, June, July), year (2017, 2018), and site (SBCP, IBSP), as the fixed effects in R Studio 1.0.136 [29]. Our global model included all main effects and two-way interactions. We chose not to examine higher level interactions because of sample size limitations and loss of degrees of freedom. We then used an information theoretic approach (AIC: Burnham and Anderson, 1998) to determine which model from our candidate set performed best using the AICcmodavg package in R [30]. Once the best model was selected we examined significant effects using a one way ANOVA or independent samples t-test for normally distributed data, and a Kruskal-Wallis test and Mann-Whitney U test for non-normally distributed data.

## Results

### Sampling effort

Two hundred thirty-three blood samples from 2017, and 160 blood samples from 2018 (393 total samples) met the inclusion criteria for this study. One hundred fifty-seven individual turtles were sampled only once, and 70 individual turtles were sampled 2–6 times. Two hundred twenty-four samples were from SBCP, and 169 were from IBSP. There were 254 samples from females, 103 from males, and 36 from turtles of unknown sex. Two hundred sixty-eight samples were from adults, 85 from subadults, and 40 from juveniles. Samples were collected in May (n = 134), June (n = 160), July (n = 98) and August (n = 1).

### Clinical pathology

Population-based and subject-based reference intervals were constructed for 393 Blanding’s turtle samples in 2017–2018 (Table 1). All significant differences between analytes are tabulated by age class (Table 2), sex (Table 3), month (Table 4), year (Table 5), and site (Table 6). Adult turtles had higher TS, H:L, Ca, Ca:P, and BA compared to juveniles, and lower total leukocytes, lymphocytes, and AST compared to subadults and juveniles. Heterophil counts were lower in adults than juveniles, and PCV was higher in juveniles than subadults (P < 0.05). Female turtles had higher H:L, Ca, Ca:P, and P, and lower CK values than males (P < 0.05). Ca was increased in July compared to both May and June, eosinophils progressively increased from May to July, and H:L, UA, AST, and P increased from May to June (P < 0.05). In 2017, turtles had higher PCV, basophils, H:L, Ca, P, and UA levels, while in 2018 total leukocytes and lymphocytes were higher (P < 0.05). Site differences include higher PCV, TS, P, UA and AST at the unmanaged IBSP location, and higher monocyte and eosinophil counts at the managed SBCP location (P < 0.05).

**Table 1:** Population-based reference interval and subject-based reference interval of complete blood count and plasma biochemical parameters in 393 apparently healthy Blanding’s turtles (*Emydoidea blandingii*). Between-animal coefficient of variance (CV_*G*_), within-animal coefficient of variance (CV_*I*_), analytical variation (CV_A_), index of individuality (Index), and reference change value (RCV).

***Population-based RI***	***N***	***Mean***	***SD***	***Median***	***Min***	***Max***	***Distribution***	***Reference Interval***	***90% CI Low Bound***	***90% CI High Bound***
PCV (%)	393	18.6	6.2	19	4	34.5	Normal	6.4–30.8	5.5–7.3	29.9–31.7
TS (g/dl)	393	3.5	1.1	3.6	0.4	7.4	Normal	1.3–5.7	1.2–1.5	5.5–5.9
Heterophils/μl	389	1473	1034	1267	0	7898	Non-normal	0–4023	0–158	3600–4554
Eosinophils/μl	389	1055	859	814	0	5173	Non-normal	0–3231	0–55	2725–4404
H:L ratio	391	0.183	0.185	0.139	0	1.78	Normal	0–0.492	0–0	0.47–0.52
Total Calcium(mg/dl)	393	10.7	5.8	9.6	1.7	41.3	Normal	3.2–26.7	3.0–3.5	24.7–28.8
Phosphorus (mg/dl)	392	3.5	1.6	3.3	0.8	11.8	Non-normal	1.3–7.1	1.2–1.4	7.2–8.2
Ca:P ratio (mg/dl)	366	3.24	1.39	3	0.28	9.53	Non-normal	1.29–5.94	1.08–1.45	5.44–6.44
Bile acids (μmol/L)	349	3.6	3.1	3	0.6	39.9	Non-normal	1–10.6	1.0–1.1	8.1–16.4
***Subject based RI***	***N***	***Mean***	***SD***	***CV***_***A***_	***CV***_***I***_	***CV***_***G***_	***Index***	***RCV***		
WBC/μl	389	20131	18809	8.2%	46%	93%	0.50	130%		
Lymphocytes/μl	389	14559	15743	8.2%	52%	108%	0.49	146%		
Monocytes/μl	389	659	904	8.2%	78%	137%	0.57	217%		
Basophils/μl	389	2371	3063	8.2%	63%	129%	0.49	176%		
Uric acid (mg/dl)	390	2.8	0.2		66%	114%	0.58	183%		
Creatine kinase (U/L)	391	897.6	1055.1		58%	117%	0.49	161%		
AST (U/L)	393	64.7	36.0		33%	56%	0.59	91%		

**Table 2:** Descriptive statistics for clinical pathology variables significantly different between age classes from 393 Blanding’s turtles (*Emydoidea blandingii*) from Lake county, IL in 2017–2018.

Analyte		n	Mean/Median	95% CI/10-90 percentile		Range		Normally distributed
				Lower bound	Upper bound	Minimum	Maximum	
PCV (%) ^a^	Adult	264	18.4	17.6	19.2	4.0	34.5	Yes
	Subadult	82	20.0	18.8	21.2	9.0	33.0	Yes
	Juvenile	37	16.6	14.9	18.4	6.5	30.0	Yes
TS (g/dl) ^b^	Adult	264	3.8	3.6	3.9	1.2	7.4	Yes
	Subadult	82	3.1	2.9	3.3	0.4	5.6	Yes
	Juvenile	37	2.7	2.4	3.0	1.0	5.0	Yes
WBC/μl ^c^	Adult	106	13029	6350	37919	2933	117040	No
	Subadult	40	18606	10151	38773	5440	101200	No
	Juvenile	79	21371	11520	54120	7413	188320	No
Heterophils/μl ^d^	Adult	261	1368	1259	1477	0	5435	Yes
	Subadult	81	1637	364	3624	0	7898	No
	Juvenile	37	1117	270	2808	112	4547	No
Lymphocytes/μl ^e^	Adult	106	7949	3140	29493	801	97143	No
	Subadult	40	13025	5769	28791	3808	87718	No
	Juvenile	79	16191	7938	40920	4522	141240	No
H:L ^f^	Adult	261	0.167	0.026	0.477	0	1.78	No
	Subadult	81	0.106	0.019	0.247	0	0.375	No
	Juvenile	37	0.096	0.023	0.258	0	0.439	No
Total Calcium (mg/dl) ^g^	Adult	268	12.1	11.3	12.9	2.3	41.3	Yes
	Subadult	82	7.7	7.0	8.5	2.3	17.7	Yes
	Juvenile	40	7.5	6.3	8.6	1.7	15.6	Yes
Ca:P ^h^	Adult	264	3.61	3.45	3.78	0.28	9.53	Yes
	Subadult	82	2.30	1.19	3.26	0.46	9.22	No
	Juvenile	37	2.44	2.14	2.74	0.65	4.45	Yes
Bile acids (μmol/L) ^i^	Adult	264	3.0	1.3	6.3	1.0	39.9	No
	Subadult	82	2.4	1.1	5.0	0.6	12.4	No
	Juvenile	37	2.6	1.1	4.9	1.0	10.1	No
AST (U/L) ^j^	Adult	106	56	35	98	19	178	No
	Subadult	40	72	39	161	21	231	No
	Juvenile	80	69	43	133	19	242	No

^a^ Subadults higher than juveniles (p = 0.017)

^b^ Adults greater than both subadult and juvenile (p<0.0001)

^c^ Adults lower than subadults (p = 0.005) and juveniles (p<0.0001)

^d^ Adults lower than subadults (p = 0.001)

^e^ Adults lower than subadults (p = 0.001) and juveniles (p<0.0001)

^f^ Adults greater than subadults (p = 0.002) and juveniles (p = 0.02)

^g^ Adults greater than both subadults and juveniles (p<0.0001)

^h^ Adults greater than both subadults and juveniles (p<0.0001)

^i^ Adults greater than both subadults and juveniles (p<0.0001)

^j^ Adults lower than subadults (p = 0.005) and juveniles (p<0.0001)

**Table 3:** Descriptive statistics for clinical pathology variables significantly different between males and females sampled from 393 Blanding’s turtles (*Emydoidea blandingii*) from Lake county, IL in 2017–2018.

Analyte		n	Mean/Median	95% CI/10-90 percentile		Range		Normally distributed	Significance
				Lower bound	Upper bound	Minimum	Maximum		
H:L	Female	245	0.167	0.026	0.439	0	1.78	No	0.03
	Male	98	0.113	0.014	0.313	0	1	No	
Total Calcium	Female	249	12.4	11.6	13.2	2.3	41.3	Yes	p<0.0001
(mg/dl)	Male	98	8.0	7.3	8.6	2.4	16.9	Yes	
Phosphorus (mg/dl)	Female	249	3.7	3.5	3.9	0.8	11.8	Yes	p<0.0001
	Male	98	3.1	2.8	3.4	0.8	8.9	Yes	
Ca:P	Female	249	3.34	2.26	4.86	0.74	8.18	No	p<0.0001
	Male	98	2.62	1.72	4.59	0.76	9.22	No	
Creatine Kinase	Female	116	565	224	1284	49	4115	No	0.003
(U/L)	Male	75	850	276	1768	83	6097	No	

**Table 4:** Descriptive statistics for clinical pathology variables significantly different between months from 393 Blanding’s turtles (*Emydoidea blandingii*) from Lake county, IL in 2017–2018.

Analyte		n	Mean/Median	95% CI/10-90 percentile		Range		Normally distributed
				Lower bound	Upper bound	Minimum	Maximum	
Eosinophils/ μl ^a^	May	133	531.41	132.59	1522.51	0	3356	No
	June	152	838.93	239.71	2221.49	0	5173	No
	July	94	1553.54	1362.77	1744.3	176	4599	Yes
H:L^b^	May	133	0.165	0.025	0.49	0	1.03	No
	June	152	0.127	0.024	0.304	0	0.702	No
	July	94	0.151	0.023	0.391	0	1.78	No
Total Calcium	May	134	8.95	4.6	17.7	2.3	33.0	No
(mg/dl) ^c^	June	153	9.30	8.60	10.10	1.7	3.0	Yes
	July	96	13.30	11.90	14.70	2.3	41.3	Yes
Phosphorus	May	134	2.80	2.60	3.10	0.8	8.6	Yes
(mg/dl) ^d^	June	153	3.60	3.40	3.90	1.2	8.9	Yes
	July	96	4.10	3.70	4.40	1.4	11.8	Yes
Uric Acid (mg/dl) ^e^	May	87	1.40	0.88	5.12	0.8	8.5	No
	June	115	1.40	0.80	1.84	0.8	4.4	No
	July	21	1.40	0.80	10.16	0.8	11.6	No
AST (U/L) ^f^	May	88	53.50	33.90	96.60	19	179	No
	June	116	68.50	43.00	126.30	30	242	No
	July	21	58.00	35.00	177.00	19	188	No

^a^ Eosinophils in May lower than June (p = 0.001) and July (p<0.0001), June lower than July (<0.001)

^b^ H:L in May higher than June (p = 0.002)

^c^ Total calcium in July higher than May (p = 0.001) and June (p<0.0001)

^d^ Phosphorous in May lower than June and July (p<0.0001)

^e^ Uric acid in turtles in May lower than June (p = 0.014)

^f^ AST in turtles in May lower than June (p<0.0001)

**Table 5:** Descriptive statistics for clinical pathology variables significantly different between year sampled from 393 Blanding’s turtles (*Emydoidea blandingii*) from Lake county, IL in 2017–2018.

Analyte		n	Mean/Median	95% CI/10-90 percentile		Range		Normally distributed	Significance
				Lower bound	Upper bound	Minimum	Maximum		
PCV (%)	2017	224	19.4	18.6	20.2	4.0	34.5	Yes	0.002
	2018	59	17.4	16.4	18.4	4.0	33.0	Yes	
WBC/μl	2017	136	14863	7243	35200	2933	101200	No	<0.0001
	2018	89	21707	10071	54560	5067	188320	No	
Lymphocytes/μl	2017	136	9585	3887	26475	801	87718	No	<0.0001
	2018	89	16191	6432	44378	2331	141240	No	
Basophils/μl	2017	136	1548	379	4075	0	15180	No	0.01
	2018	89	2187	634	7060	256	35781	No	
H:L	2017	221	0.167	0.035	0.415	0.01	1.78	No	0.002
	2018	158	0.097	0.013	0.339	0	1	No	
Total Calcium	2017	224	12.3	11.5	13.1	2.7	41.3	Yes	p<0.0001
(mg/dl)	2018	159	7.0	3.8	15.8	1.7	27.1	No	
Phosphorus	2017	224	4.0	3.8	4.2	1.1	11.8	Yes	p<0.0001
(mg/dl)	2018	159	2.7	2.5	2.9	0.8	7.0	Yes	
Uric Acid (mg/dl)	2017	135	1.4	0.9	5.0	0.8	11.6	No	0.006
	2018	89	1.4	0.8	1.8	0.8	4.4	No	

**Table 6:** Descriptive statistics for clinical pathology variables significantly different between sites from 393 Blanding’s turtles (*Emydoidea blandingii*) from Lake county, IL in 2017–2018.

Analyte		n	Mean/Median	95% CI/10-90 percentile		Range		Normally distributed	Significance
				Lower bound	Upper bound	Minimum	Maximum		
PCV (%)	CSB	216	17.3	16.5	18.0	4.0	28.0	Yes	p<0.0001
	IBSP	167	20.2	19.2	21.2	4.0	34.5	Yes	
TS (g/dl)	CSB	216	3.3	3.1	3.5	0.4	7.1	Yes	p<0.0001
	IBSP	167	3.8	3.6	3.9	1.2	7.4	Yes	
Monocytes/μl	CSB	147	554	125	1632	0	9416	No	p = 0.012
	IBSP	77	387	41	1243	0	4048	No	
Eosinophils/μl	CSB	215	972	238	2346	0	5173	No	p<0.0001
	IBSP	164	657	187	1744	0	4404	No	
Phosphorus	CSB	216	3.3	3.1	3.5	0.8	7.9	Yes	p = 0.029
(mg/dl)	IBSP	167	3.7	3.4	3.9	0.8	11.8	Yes	
Uric Acid (mg/dl)	CSB	147	1.4	0.8	2.2	0.8	11.6	No	p = 0.003
	IBSP	76	1.4	0.9	5.1	0.8	6.8	No	
AST (U/L)	CSB	147	58	34	107	19	242	No	p = 0.001
	IBSP	78	74	44	149	35	231	No	

## Discussion

Hematologic and plasma biochemical parameters are key components of routine health monitoring in many species [13]. These parameters are most valuable for health assessment when species-specific reference values are available for comparison [30]. This study utilized 393 blood samples from two active seasons to establish baseline hematology and plasma biochemistry values for the Lake Plain Blanding’s turtle, and is the first study to evaluate clinical pathology in this species. It is important to recognize that natural variation in reptilian species can influence the sensitivity of RI classification, and thus may be species-specific. PCV, TS, heterophils, eosinophils, H:L ratio, total Ca, P, Ca:P ratio, and bile acids were assigned population-based RIs, as more variation occurred intra-individually for these parameters. WBC, lymphocytes, monocytes, basophils, UA, CK and AST, were alternatively assigned subject-based RIs, as more variation occurred inter-individually. The reference intervals generated in this study can be utilized as a diagnostic aid for future health assessments and conservation efforts specific to the Lake Plain population, as well as other populations of this imperiled species.

Each demographic variable observed in this study drove multiple health parameters. Subadults presented with higher PCV than juveniles, while adults presented with lower total leukocytes and lymphocytes compared to juveniles and subadults, lower heterophils than subadults, and higher H:L compared to both juveniles and subadults. There are two methodical factors that most likely contribute to the significant hematologic differences in regards to age class: blood collection site and the Leukopet method of leukocyte analysis. It is known that the subcarapacial sinus has been associated with hemodilution due to presence of interwoven lymphatics [31–34], which can lower volume percentages of cellular components and enzyme activities of samples [35–36]. Additionally, venipuncture in juveniles was technically more challenging and prone to visual hemodilution due to both technical skill and needle size. Due to the way the Leukopet calculation is performed, species with low heterophil counts or samples with artifactually lower heterophil count due to hemodilution, have increased error [37–39]. In turn blood collection from the subcarapacial sinus evaluated with the Leukopet method of analysis could significantly skew analyte measures. It should be recognized however that the Leukopet is one of few methods available to estimate leukocyte profiles of non-mammalian species due to their nucleated erythrocytes.

Adult turtles had higher TS, Ca, Ca:P and BA, while juvenile and subadult turtles had higher AST. Several of these differences can be attributed to reproductive physiology. Mature female chelonians are known to exhibit hypercalcemia and hyperproteinemia during vitellogenesis [12–13, 37] explaining increased TS, Ca, and Ca:P in adults. Moderate hepatic lipidosis in chelonians during vitellogenesis is not abnormal [40], and a reasonable explanation for higher bile acids in adults. As a metabolite of cholesterol, post-prandial lipemia is also known to increase bile acid values in falcons (*Falco peregrinus*) [41] and green iguanas [42–43] thus differences in diet availability or feeding behavior between age classes should also be considered. Unfortunately, this fasting factor is uncontrollable in a free-ranging population of Blanding’s turtles, independent of age. In lieu of these explanations, all BA were below 5 μmol/L, thus the differences between age classes likely represent a statistical significance rather than a biological significance. Elevated AST activity is not liver-specific but can be indicative of hepatocellular disease in reptiles [37, 44–45]. In the absence of liver disease, increases are associated with hyperactivity, aggression between individuals [13, 19, 46–47], and growth [33] which are characteristics that may be more prominent in juveniles and subadults.

H:L was affected by both age class and sex, being highest in adult, female turtles. As increased H:L is recognized as a measure of stress in reptiles [37, 48–49], metabolic stress due to vitellogenesis may induce changes in heterophil and lymphocyte populations. Higher Ca, P, and Ca:P in females compared to males can also be explained by reproductive physiology, while higher CK in males compared to females is better explained by behavior. In reptiles higher CK is associated with muscle damage, systemic infection, exertion during physical handling [37] and increased metabolic rates [50]. Higher values for muscle damage markers have been previously identified in male turtles [19, 51], and our findings are consistent with these studies.

Several health parameters were also driven by month. Eosinophils progressively increased from May to July, which is a trend that has also been observed in box turtles [15] and gopher tortoises [52]. It is suggested that increased exposure to parasites or other immune stimulating factors occurs later in the sampling season, accounting for this observation. H:L was higher in May than June, possibly driven by increased heterophil concentrations. Heterophil concentrations have previously been reported to increase during summer months and decrease during brumation in turtles [37, 52]. The sampling periods for this study did not include brumation, however, future studies should investigate this parameter earlier in spring and later in fall. P, UA, and AST were observed to increase from May to June. As a protein catabolite post-prandial samples are known to increase UA in reptiles [53] and birds [54]. Diet availability or feeding behaviors revolving around protein sources therefore may vary with month. The increase in AST suggests behaviors of hyperactivity, aggression and/or growth may also vary with month [13, 31, 46–47]. Although month alone seems an unlikely driver of renal function, increased P, UA and AST, have been associated with renal insult and ultimately a poorer plane of health [53, 55–57]. If previously presented speculations are not responsible for the trend in these parameters, it can be suggested that renal challenge varies with month. General etiologies of renal disease in reptiles that have been presented and should be considered are toxicities, infectious organisms, nutritional diseases, metabolic disorders, degenerative processes, and neoplasia [57].

Turtles in 2017 had lower total leukocyte and lymphocyte counts, but higher PCV, basophils, H:L, Ca, P, and UA. Annual hematologic differences may best be explained by environmental factors. Previous studies have found higher PCV in chelonians during both drier months [13, 51], and warmer months [50, 58], suggesting humidity and temperature influence longitudinal hematologic differences. Differences in PCV were also observed to vary annually over a three-year period in eastern box turtles, possibly due to similar mechanisms in a temperate environment [15]. Yearly variation in exposure to disease, stress and other immune stimulating factors should be considered a plausible explanation for differences in basophil and H:L measures, warranting further investigation. Decreased Ca and P in 2018 may be explained by intentional avoidance of sampling gravid females this year (per advised by LCFPD management to encourage nesting success) or a decrease in protein-bound total calcium. Active ionized calcium is a recommended parameter to be monitored in future assessments as it isn’t influenced by protein. True calcium deficiencies can cause havoc for both the metabolic and reproductive systems of reptiles [59].

To complete the assessment of demographic drivers, analytes were compared between the actively managed SBCP site and unmanaged IBSP site. Interestingly, two parameters that did not differ between sites were H:L and total calcium, suggesting populations may have similar stressors and reproductive character. PCV, TS, UA, AST and phosphorous were all significantly greater in IBSP turtles, while eosinophils and monocytes were greater in SBCP turtles. There were several phlebotomists whom collected samples utilized in this study, however each site had a different principle sample collector each year. It is possible that during both years the principal phlebotomist at SBCP was more likely to get a lymph contaminated sample, explaining higher PCV/TS values at IBSP. If former nutritional and reproductive justifications are not responsible for site differences, two physiologic systems appear to be superiorly challenged at IBSP relative to SBCP. Higher PCV, TS, and UA, suggest threats to hydration may be more prominent at IBSP. Higher P, UA, and AST, suggest renal disease may also be of greater threat to turtles at IBSP. These observations beg the conclusion that currently implemented conservation actions at SBCP may be benefiting turtle health.

On the contrary, finding higher eosinophils and monocytes at SBCP indicates there may be increased exposure to parasites. A single subadult female from SBCP died from heavy parasitism in early August 2018, thus it is evident that exposure to parasites has occurred at this site and has potential for mortality. Due to the delayed sexual maturity of this species and limited population of adult individuals, the loss of any individual, especially one with potential to reproduce, can significantly restrict population growth rates [60]. Research regarding parasite exposure and effects is warranted, and may be a possible target of future conservation management at this location.

This study described numerous aspects of the host clinical pathology, but the findings should be put in the context of certain unique limitations of sample collection and analysis that have potential to effect both leukocyte and biochemical analytes [24]. There is respectful concern that some individuals would be classified as unhealthy based on absolute value alone. Outliers were included in population analysis to serve as a reference for clinically healthy animals that report erroneous clinical pathology values. Values should always be interpreted in respect to clinical presentation and if possible should be evaluated in serial. Venipuncture using the subcarapacial sinus [33–36], unknown fasting states [42–43, 53–54], time between capture and sample collection [61], delayed sample processing [62–65], and the Leukopet method of leukocyte analysis, all have potential for significant effects on leukocyte and biochemical profiles and should be considered for some of the extreme absolute values. Hemodilution associated with the subcarapacial sinus can falsely lower several clinical pathology values [35,36], thus reported minimums such as those for heterophils (0%), Ca (1.7), P (0.8) and PCV (4%) are possibly in part due to lymph artifact. The Leukopet method can falsely elevate total leukocyte counts in the presence of low heterophils [66], thus reported WBC maximums >100,000 are possibly due to method of analysis rather than true pathologic disease. Natt and Herick’s, opposed to the Leukopet, has proven to be more precise in Galapagos tortoises (Chlonoidis spp) [62], and comparing this method in the lymphocyte-dominant Blanding’s turtle may better determine if significant hematology represents true biological implication or an analytical limitation. Ultimately, all of these limitations have potential to significantly alter hematologic and biochemical measures and need to be considered when evaluating health.

## Conclusion

This study defines reference intervals for 16 hematologic and plasma biochemical analytes, characterizes demographic and temporal drivers of clinical pathology, and describes differences in bloodwork between managed and unmanaged sites. Conservation efforts have been successfully adopted for Lake County’s Blanding’s turtle population, however this study is the first to analyze effects on turtle health. The established reference intervals can serve as a baseline for future health assessments of this population and others. The evaluation of age class, sex, month, and year has exposed threats to physiologic systems and warrants further investigation of sensitivity and specificity of clinical pathology parameters to better assist in diagnosis of disease. The differences in bloodwork between two sites has identified concerns at each location which can be utilized to steer strategies to conserve the Blanding’s turtle. Future conservation management for this population should consider the observations reflected while continuing health assessments to monitor effects of management action on this imperiled species.

## Supporting information

S1 File Data set. (XLSX)
